# ^11^C-Choline and FDG PET/CT Imaging of Primary Cholangiocarcinoma: A Comparative Analysis

**Published:** 2015

**Authors:** Chanisa Chotipanich, Chetsadaporn Promteangtrong, Anchisa Kunawudhi, Rawisak Chanwat, Thaniya Sricharunrat, Savitree Suratako, Paramest Wongsa

**Affiliations:** 1 National Cyclotron and PET Centre, Chulabhorn Hospital, Thailand; 2 Surgery Department, Chulabhorn Hospital, Thailand; 3 Pathology Department, Chulabhorn Hospital, Thailand

**Keywords:** Cholangiocarcinoma, Choline, FDG, PET/CT, Radiotracer

## Abstract

**Objective(s):**

This study aimed to compare the diagnostic values of ^11^C-choline and ^18^F-fluorodeoxyglucose (^18^F-FDG) positron emission tomography/computed tomography (PET/CT) in patients with cholangiocarcinoma (CCA).

**Methods:**

This prospective study was conducted on 10 patients (6 males and 4 females), aged 42-69 years, suspected of having CCA based on CT or magnetic resonance imaging (MRI) results. ^11^C-choline and ^18^F-FDG PET/CT studies were performed in all patients over 1 week. PET/CT results were visually analyzed by 2 independent nuclear medicine physicians and quantitatively by calculating the tumor-to-background ratio (T/B).

**Results:**

No ^11^C-choline PET/CT uptake was observed in primary extrahepatic or intrahepatic CCA cases. Intense ^18^F-FDG avidity was detected in the tumors of 8 patients (%80). Two patients, who were ^18^F-FDG negative, had primary extrahepatic CCA. Ki-67 measurements were positive in all patients (range; 14.2%-39.9%). The average T/B values of ^11^C-choline and ^18^F-FDG were 0.4±0.2 and 2.0±1.0 in all cases of primary CCA, respectively; these values were significantly lower for ^11^C-choline (P<0.005). Both FDG and ^11^C-choline PET/CT detected metastatic CCA foci in all 8 patients (two patients had no metastases).

**Conclusion:**

As the results suggested, primary CCA lesions showed a poor avidity for ^11^C-choline, whereas ^18^F-FDG PET/CT was of value for the detection of most primary CCA cases. In contrast to primary lesions, metastatic CCA lesions showed ^11^C-choline avidity.

## Introduction

Cholangiocarcinoma (CCA) is a form of malignant liver tumor, arising from cholangiocytes. This condition has an incidence of 10-15% among all primary liver and bile duct malignancies and is the second most common primary liver cancer after hepatocellular carcinoma (HCC) (1).

According to the statistics published by The National Cancer Institute of Thailand in 2009, liver and bile duct cancers, with an incidence rate of 7.1%, rank the third (12.4%) and fifth (4%) most common cancers in males and females, respectively (2). Also, a higher incidence of CCA has been reported among populations in the northeastern parts of Thailand, compared to other regions (3). For instance, in the northeastern province of Khon Kaen, CCA accounts for 71% of primary liver and bile duct cancers, probably due to the endemic spread of opisthorchiasis in this region (4).

Prognosis of CCA treatment depends on the location, stage, and the presence of metastases. Surgical treatment is the only curative therapeutic option in patients with localized disease; however, less than 20% in early stage CCA (5-7). Accurate tumor staging is therefore crucial for treatment planning in patients with CCA.

At present, diagnosis and staging of CCA are performed by ultrasound (US), computed tomography (CT), and magnetic resonance imaging (MRI) (6-7), which provide information regarding tumor extension, bile duct enlargement, and the size of nearby lymph nodes. However, these modalities are limited in differentiating between benign and malignant lesions. In fact, up to 10% of malignant-appearing biliary strictures appear benign during surgical explorations. Furthermore, up to 50% of patients with CCA present with metastatic lymphadenopathy, which is often missed on conventional preoperative size-dependent imaging (8). Therefore, invasive methods have been recommended to achieve further pathological confirmation.

These limitations have led to the evaluation of metabolic imaging by positron emission tomography/CT (PET/CT) for a more accurate assessment of CCA. ^18^F-fluorodeoxyglucose (^18^F-FDG) imaging is a widely accepted non-invasive tool, which provides molecular information regarding cellular glucose metabolism in most cancers. This modality has a confirmed high value in the diagnosis, staging, treatment planning, and prognosis of malignancies.

The sensitivity of ^18^F-FDG imaging for the evaluation of CCA ranges between 69.2% and 95%, and its specificity varies between 66.7% and 100% (9-11). The accuracy of ^18^F-FDG imaging depends on the location, growth rate, and pathologic characteristics of CCA. The sensitivity of this technique is reported to be higher in nodular CCA, compared to the infiltrating type; however, negative results have been reported in mucinous adenocarcinoma (9). For the evaluation of metastatic lymph nodes, ^18^F-FDG PET/CT has a sensitivity of only 12-43% with a high specificity of 96-100%, whereas for the detection of distant metastases, the sensitivity and specificity are 94-100% and 100%, respectively (9, 12-13).

Considering the relatively low performance indices of ^18^F-FDG PET for the evaluation of infiltrating extrahepatic CCA and mucinous adenocarcinoma and given the low sensitivity of this modality for the diagnosis of metastatic lymph nodes, application of other PET radiopharmaceuticals has been advocated. While ^11^C-choline targeting lipid metabolism is a potentially interesting approach for the diagnosis and staging of CCA, its applicability for this specific clinical indication has not been previously assessed. The purpose of the present study was to evaluate the diagnostic value of ^11^C-choline PET/CT imaging and compare it with ^18^F-FDG PET/CT in the diagnosis and staging of CCA.

## Methods

This study was approved by the Chulabhorn Institutional Review Board. Written informed consents were obtained from all the patients.

### Patients

This prospective study included patients undergoing both ^11^C-choline and ^18^F-FDG PET/CT imaging for the initial staging of CCA at the National Cyclotron and PET Centre of Chulabhorn Hospital between October 2011 and September 2012.

Ten patients (6 males and 4 females), aged 42 to 69 years (mean age: 54.1 years), suspected of having CCA based on CT or MRI results, were evaluated prior to surgery or biopsy after obtaining informed consents. ^11^C-choline and ^18^F-FDG PET/CT studies were performed for all patients within a 1-week interval. Diagnosis of primary tumor was histologically confirmed in all patients, whereas the detection of lymph node involvement and distant metastasis was based on histopathology and/or contrast-enhanced CT imaging.

### PET/CT imaging procedures

Patients fasted for 6 hours prior to the FDG PET/CT scanning, performed using Siemens Biograph 16 PET/CT scanner in the 3D mode. Imaging was performed 90 minutes after the intravenous injection of 5 MBq/kg ^18^F-FDG and 5 minutes after the intravenous injection of 6 MBq/kg ^11^C-choline. Plasma glucose level was determined prior to the ^18^F-FDG PET/CT study.

For the ^11^C-choline PET studies, contrast-enhanced CT was performed, following the intravenous administration of the contrast medium. Image acquisition was performed from the skull base to the proximal thigh 3 minutes/per bed position with 4 iterations, 8 subsets, matrix size of 168, zoom 1, and a Gaussian filter with Full Width at Half Maximum (FWHM) of 5.0.

### Data analysis

PET/CT results were analyzed by 2 independent and experienced nuclear medicine physicians, unaware of the clinical data, CT/MRI findings, or histopathological results at the time of review.

For the visual analysis, areas of non-physiologically increased uptake were defined as malignant lesions by consensus. For the quantitative analysis, the T/B ratio was calculated by volumetrically measuring the mean standardized uptake value (SUVave) of the tumor over the volume of interest in PET, dividing it by the average SUVave of the normal parenchyma of the right liver lobe (the left lobe was used in case of any lesions in the right lobe), calculated from averaging the measurements over three regions of 2 cm^3^ sphere volume.

### Reference standard

Histopathological diagnosis was used as the reference standard. Since the histological confirmation of all metastatic lesions was not technically feasible, lesions showing focal increased tracer uptake beyond the physiological range, with a corresponding CT abnormality, were also defined as true positive sites of distant metastasis.

### Statistical analysis

The diagnostic accuracy and sensitivity of both radiotracers were calculated. The quantitative T/B parameter was measured for all the primary sites and was compared between the two radiotracers by Mann-Whitney U and Wilcoxon signed-rank tests, using SPSS version 11.5. P-value less than 0.05 was considered statistically significant.

## Results

Ten patients were enrolled in this study between October 2011 and September 2012. The mean time interval between the ^18^F-FDG and ^11^C-choline studies was 2.1 days and the mean interval between PET/CT and surgery was 13.7 days.

Histology indicated well differentiated CCA in 6 patients, moderately differentiated CCA in 3 patients, and poorly differentiated CCA in 1 patient. The Ki-67 index (%) ranged between 14.3% and 39.9% (mean: 25.9%±8.7%). Of all patients, 2 cases (20%) had a single lesion and the remaining had more than 2 lesions including bone and lymph node metastases.

**Figure 1:**
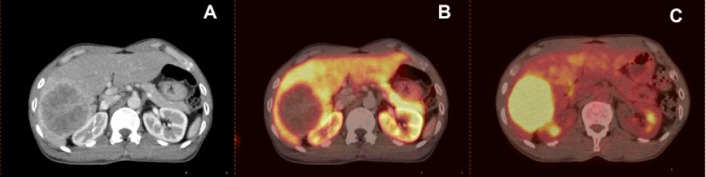
A 57-year-old man with well differentiated intrahepatic CCA. A) Transaxial contrast-enhanced CT shows a right lobe liver mass. B) Transaxial ^11^C-choline PET/CT shows no significant uptake in the lesion (T/B ratio= 0.23). C) Transaxial ^18^F-FDG PET/CT shows increased uptake in the right lobe liver mass (T/B ratio= 2.05)

**Figure 2:**
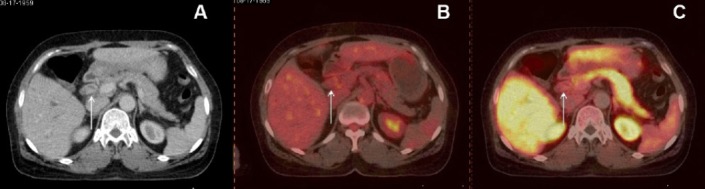
A 52-year-old woman with well differentiated extrahepatic CCA. A) Transaxial contrast-enhanced CT shows an intraluminal, irregular, enhancing soft tissue lesion. B) Transaxial ^11^C-choline and C) transaxial ^18^F-FDG PET/CT images show no significant uptake in the lesion with T/B ratios of 0.6 and 0.78, respectively

**Figure 3:**
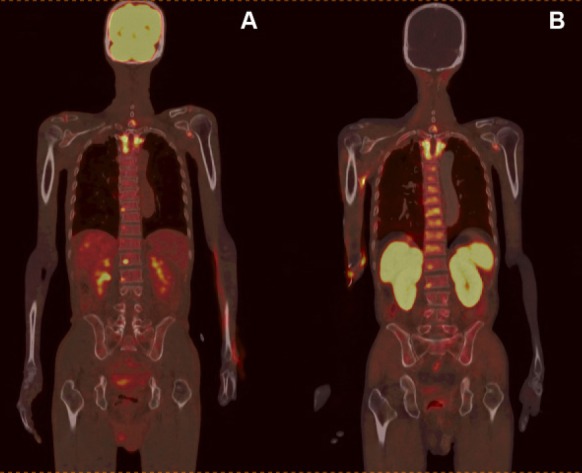
Bone metastases in the upper thoracic spine detected by both A) ^18^F-FDG and B) ^11^C-choline PET/CT

Patient characteristics are shown in Table 1 and the results are summarized in Tables 2 and 3. Figures 1-3 show cases of CCA lesions detected by only ^18^F-FDG, lesions detected by none of the tracers, and detected bone metastases by both tracers, respectively.

**Table 1:** Patient characteristics

Characteristics	Number (%)
Sex	
Male	6 (60)
Female	4 (40)
Age (years)	
Mean	54.1
Range	42–69
Histology	
Well differentiated	6 (60)
Moderately differentiated	3 (30)
Poorly differentiated	1 (10)
Ki-67	
≤ 15%	1 (10)
16-30%	6 (60)
>30%	3 (30)

**Table 2:** Disease characteristics and PET/CT findings of each patient

Patient No.	Age	Sex	Primary location	Differentiation	Ki-67(%)	^11^C-choline	T/B ratio	^18^F-FDG	T/B ratio
1	61	M	Intrahepatic	WD	18.9	Neg.	0.48	Pos.	1.72
2	65	F	Intrahepatic	WD	35.5	Neg.	0.41	Pos.	4.16
3	57	M	Intrahepatic	WD	32.7	Neg.	0.23	Pos.	2.05
4	55	M	Intrahepatic	WD	15.2	Neg.	0.26	Pos.	1.51
5	53	F	Intrahepatic	MD	29.2	Neg.	0.35	Pos.	2.38
6	43	F	Intrahepatic	MD	14.3	Neg.	0.57	Pos.	1.3
7	69	M	Intrahepatic	MD	28.2	Neg.	0.1	Pos.	2.57
8	44	M	Intrahepatic	PD	39.9	Neg.	0.48	Pos.	2.34
9	52	F	Extrahepatic	WD	20.3	Neg.	0.6	Neg.	0.78
10	42	M	Extrahepatic	WD	24.6	Neg.	0.56	Neg.	0.92

WD: well differentiated; MD: moderately differentiated; PD: poorly differentiated; Neg.: negative; Pos.: positive

**Table 3:** Details of ^18^F-FDG and ^11^C-choline uptake in metastatic lesions

Patient No.	Location of metastasis	Number of lesions	^11^C-choline	^18^F-FDG
1	Abdominal LN	4	Pos.	Pos.
2	Intra-abdominal LN	2	Pos.	Pos.
3	-		-	-
4	Bone	7	Pos.	Pos.
5	Diaphragmatic node	2	Pos.	Pos.
6	Intra-abdominal LN	2	Pos.	Pos.
7	Bone	21	Pos.	Pos.
8	Intra-abdominal LN	8	Pos.	Pos.
9	-		-	-
10	Mediastinal LN	5	Pos.	Pos.

LN: lymph node; Neg.: negative; Pos.: positive

### Primary CCA lesions

Ten intrahepatic lesions were detected in 8 patients by ^18^F-FDG PET/CT imaging, while none of the lesions were detected by ^11^C-choline PET/CT. Two patients had a primary extrahepatic lesion, which was not detected by either ^18^F-FDG or ^11^C-choline PET/CT. These two false-negative FDG lesions included a 1.1 cm well-differentiated adenocarcinoma at the distal common bile duct and a 1.5 cm well-differentiated hilar CCA. The sensitivity and accuracy of ^18^F-FDG PET/CT for the detection of primary CCA were 80%.

The T/B ratio for ^11^C-choline was estimated at 0.4±0.2 (range: 0.1-0.57), which was significantly lower than that of ^18^F-FDG (2.0±1.0) (range: 0.78-4.16, P=0.008). Further analysis was carried out according to histological subtypes. For well differentiated tumors, the T/B ratio in ^11^C-choline was significantly lower than observed in ^18^F-FDG (0.42±0.15 vs. 1.86±1.23) (*P*=0.004). For moderately differentiated tumors, the T/B ratio in ^11^C-choline was lower than that reported in ^18^F-FDG, although the difference was not statistically significant (0.34±0.24 and 2.08±0.69, respectively) (*P*=0.05).

The mean T/B ratio of ^18^F-FDG in moderately differentiated CCA was higher than that reported in well-differentiated ones. In contrast, the mean T/B ratio of ^11^C-choline in moderately differentiated CCA was lower than that observed in well-differentiated tumors; however, these differences were not statistically significant (P>0.05).

### Metastatic CCA

As the results indicated, 2 patients had no metastases. Both FDG and ^11^C-choline PET/CT detected metastatic CCA foci in all 8 patients with disseminated disease and all distant sites in the same patient being of the same type, lymph node metastases (6 patients, n=23) and bone metastases (2 patients, n=28). All metastatic lesions at all sites could be detected by both tracers via visual analysis. The T/B ratio of metastatic site was 0.77±0.23 for ^11^C-choline and 2.98±0.69 for ^18^F-FDG. It should be mentioned that in ^11^C-choline studies, even though we could detect the lesions by visual analysis, the T/B ratio of the metastatic site was < 1, given the high liver activity.

## Discussion

Elevated levels of choline, phosphocholine, and phophatidylcholine have been detected in many types of cancer via MR spectroscopy and biochemical techniques, correlated with the increased synthesis rate of cellular membrane phospholipid. Choline tracers including ^11^C-choline and ^18^F-fluorocholine have been developed with clinical data suggesting favorable imaging characteristics in many cancers with low ^18^F-FDG avidity such as brain, prostate, and liver cancers (14-16).

So far, there have been no studies reporting the use of ^11^C-choline PET/CT for the evaluation of patients with CCA (17). A single study by Talbot et al. (14) compared the diagnostic values of ^18^F-fluorocholine and ^18^F-FDG for detecting and staging HCC in 81 patients with chronic liver disease and suspicious liver nodules. In 4 patients, the findings were compatible with CCA and 3 of these patients were positive on both PET/CT examinations. In a lesion-based approach, of 11 sites compatible with CCA in the liver, 10 were detected by ^18^F-FDG and only 6 were identified by ^18^F-fluorocholine.

Previous studies have shown that the sensitivity of ^18^F-FDG for the detection of primary CCA ranges between 55% and 84% (12-13, 18-20). In one meta-analysis (21), the pooled sensitivity and specificity for primary CCA tumor detection were 82% and 75% for PET/CT, 95% and 83% for intrahepatic CCA, and 76% and 74% for extrahepatic CCA, respectively. In the present study, of 10 patients with suspected CCA undergoing surgery, primary tumor was ^18^F-FDG positive in 80% of cases with intrahepatic disease (sensitivity: 80%); the results were in agreement with the findings of previous reports. On the other hand, two extrahepatic CCA tumors failed to show the uptake of either of the tracers; this discrepancy may be due to differences in the growth patterns (9, 22).

Extrahepatic CCA presents as an infiltrating tumor without any evidence of tumor mass formation, which might be necessary for providing a sufficient positron emission signal (13). Anderson et al. reported that FDG imaging has a low sensitivity (18%) in infiltrative CCA tumors (9). Tumor differentiation appears to be an important factor for the uptake of ^18^F-FDG and ^11^C-choline. The T/B ratio of ^18^F-FDG was higher than that of ^11^C-choline in well differentiated tumors. Furthermore, ^18^F-FDG showed a higher avidity for moderately differentiated tumors, compared to the well differentiated ones. Two false-negative FDG lesions in this study were both small, well-differentiated lesions (tumor size: 1.1 and 1.5 cm).

None of the primary lesions, either intrahepatic or extrahepatic, could be detected by ^11^C-choline PET/CT. More interestingly, all intrahepatic lesions detected by ^11^C-choline had a T/B ratio of < 1, indicating that the choline activity of tumor is less than normal liver parenchyma activity. This may suggest the lower membrane lipid phosphorylation in primary CCA, compared to normal tissues.

The present findings are similar to those reported by Nejjari et al. (23), who showed lower fluorocholine uptake in hepatic neuroendocrine metastases, compared to normal liver in mouse xenograft models. Nejjari et al. hypothesized that these findings may be related to the tumor microenvironment in the liver or the competitive uptake between the normal liver tissue and the tumor. We hypothesize that the lower detection rate in various types of liver lesion (e.g., CCA, HCC, and metastases) is mainly due to the higher physiologic choline metabolism in normal liver parenchyma.

These findings may have a high clinical significance, explaining the potential false-negative MR spectroscopy studies, by using choline ratio, aiming to differentiate between CCA and other malignant or benign liver lesions (24). Further studies at both chemical and cellular/molecular levels, as well as MR spectroscopy, should lead to improved diagnostic potential with respect to circulating tumor markers and imaging.

Contrary to primary tumors, all metastatic lesions were detected by both tracers. This could be due to the increased aggressiveness of metastatic lesions and/or the presence of a different microenvironment in various organs. The high physiologic choline metabolism in normal liver could be also a cause of failure in detecting primary lesions, while metastatic sites in lymph nodes and bones were detected due to the higher regional T/B ratio (25).

One caveat is the interpretation of ^11^C-choline uptake in mediastinal lymph nodes as it may not necessarily due to metastasis but could also represent reactive lymph nodes. Findings of various studies have cautioned researchers about the evaluation of mediastinal lymph nodes with choline PET, since non-specific mediastinal node choline activity can be observed without being significantly related to tumor characteristics (15, 17, 26, 27).

Lack of histological confirmation of mediastinal lesions is among the limitations of this study. The current study was also constrained by the small number of patients, which might have decreased the study’s statistical power. Further studies are required to determine the clinical usefulness on a larger scale.

Although the pathogenesis of the development and progression of CCA remains unclear, several molecular alterations related to the proliferation, growth, apoptosis resistance, and invasiveness of CCA have been reported (28). Additionally, in our study, all except for one case of primary CCA had Ki-67 index of ≥ 15%. Ki-67 protein is an excellent marker for the determination of proliferating cells in human and animal neoplasms (29, 30). Considering the poor performance of ^11^C-choline PET in the evaluation of primary CCA, other non-FDG tracers, which target different metabolic pathways, should be investigated.

## Conclusion

Despite the limitations of this study, our data suggest that ^11^C-choline PET/CT is not useful for the detection of primary CCA, although it can be potentially used for confirming the presence or absence of metastatic CCA lesions. ^18^F-FDG PET/CT was a better imaging agent for CCA, especially for intrahepatic CCA; however, it had a poor diagnostic value in extrahepatic primary CCA lesions. Further research on large populations is required to evaluate the value of ^18^F-FDG and the use of additional PET agents.
